# Association between risk factors and 1-year mortality of physical and/or cognitive components of post-intensive care syndrome in patients with sepsis

**DOI:** 10.1097/MD.0000000000048425

**Published:** 2026-04-17

**Authors:** Michiteru Miyazaki, Hirokuni Tagaya, Kazumasa Miida, Hidenori Kariya, Yuichi Kataoka, Masayasu Arai

**Affiliations:** a Department of Rehabilitation, Kitasato University Hospital, Sagamihara, Kanagawa, Japan; b Graduate School of Medical Sciences, Kitasato University, Sagamihara, Kanagawa, Japan; c School of Allied Health Sciences, Kitasato University, Sagamihara, Kanagawa, Japan; d Department of Emergency and Critical Care Medicine, School of Medicine, Kitasato University, Sagamihara, Kanagawa, Japan; e Division of Intensive Care Medicine, Department of Research and Development Center for New Medical Frontiers, School of Medicine, Kitasato University, Sagamihara, Kanagawa, Japan.

**Keywords:** Barthel index, delirium, long-term outcome, Mini-Mental State Examination, post-intensive care syndrome, sepsis

## Abstract

Survivors of sepsis are at high risk of developing post-intensive care syndrome (PICS), which encompasses physical, mental, and cognitive impairments after critical illness. Among these domains, physical and cognitive impairments have been linked to increased long-term mortality; however, their specific associations with mortality among patients with sepsis remain unclear. This study aimed to examine the relationship between PICS and subsequent survival, with a particular focus on physical and cognitive domains. In this retrospective cohort study, we included patients with sepsis who were admitted to the intensive care unit (ICU) for ≥ 48 hours between 2014 and 2021. Demographic and clinical data from admission, physical and cognitive impairments 1 month after ICU discharge; and 1-year survival were obtained from electronic medical records. Binary logistic regression analyses were performed to identify independent risk factors for the development of the physical and/or cognitive components of the PICS and for each individual domain. Cox proportional hazards models were used to determine the independent factors associated with 1-year mortality. Among the 210 eligible patients, 99 (47%) developed the physical and/or cognitive components of PICS, and 43 (20%) died within 1 year after ICU discharge. Physical impairment was present in 96 patients (45%) and cognitive impairment in 59 patients (28%). In the logistic regression analysis adjusted for covariates, increasing age (odds ratio [OR] 1.05, 95% confidence interval [CI] 1.02–1.09; *P* < .001), longer ICU stay (OR 1.09, 95% CI 1.01–1.17; *P* = .031), and prolonged delirium (OR 1.41, 95% CI 1.19–1.67; *P* < .001) were independently associated with the development of physical and/or cognitive components of PICS. In multivariable Cox analyses, the presence of these components was independently associated with higher 1-year mortality (hazard ratio 1.51, 95% CI 1.06–2.15; *P* = .021). When analyzed separately, physical impairment was associated with increased 1-year mortality, whereas cognitive impairment was not. Increasing age, prolonged delirium during ICU stay, and longer ICU stay were independently associated with the development of the physical and/or cognitive components of PICS. Among sepsis survivors evaluated at 1 month after ICU discharge, the presence of these components (particularly physical impairment) was independently associated with increased 1-year mortality.

## 1. Introduction

Sepsis is a life-threatening organ dysfunction caused by abnormal host response to infection.^[1]^ Reporting sepsis epidemiology accurately is challenging because of evolving definitions, variations in reporting, demographic disparities, and discrepancies in healthcare resources,^[2,3]^ however, estimates of sepsis cases still vary widely, ranging from 19 to 48.9 million annually.^[3,4]^ Mortality among critically ill patients in the intensive care unit (ICU) has decreased with the continued development of tools and techniques for providing life support,^[5]^ and short-term survival among patients with sepsis has improved in recent years.^[6–8]^ As a result, patients with sepsis frequently experience new symptoms, long-term disabilities,^[9]^ worsening preexisting health conditions, and an increased risk of death following hospitalization.^[10]^ Considering these poor outcomes, the Society for Critical Care Medicine has defined post-intensive care syndrome (PICS) as new or worsening cognitive, physical, and mental health impairments that persist beyond acute hospitalization.^[11]^ PICS can develop during ICU stay or after ICU/hospital discharge, thereby impairing the long-term prognosis of affected patients,^[11,12]^ and sepsis survivors are particularly at high risk for this syndrome.^[13]^ The incidence rate of PICS, including in patients with sepsis, has been reported to be 50 to 70% 6 months after ICU discharge.^[14–16]^ PICS at 3 months was related to a significant increase in 2-year mortality in patients with sepsis; however, the study reported that it was not an independent predictor in the multivariate analysis,^[16]^ and whether the occurrence of PICS in patients with sepsis is independently associated with mortality has not been fully investigated. Among the 3 domains of PICS, physical impairment and cognitive impairment have been reported to be associated with increased long-term mortality.^[17]^ However, the domains associated with mortality in patients with sepsis have not been clearly elucidated. Therefore, this study focused on the physical and cognitive domains of PICS, with the objective of examining how PICS occurrence relates to subsequent survival.

## 2. Participants and methods

### 2.1. Setting and study participants

**Figure 1.**
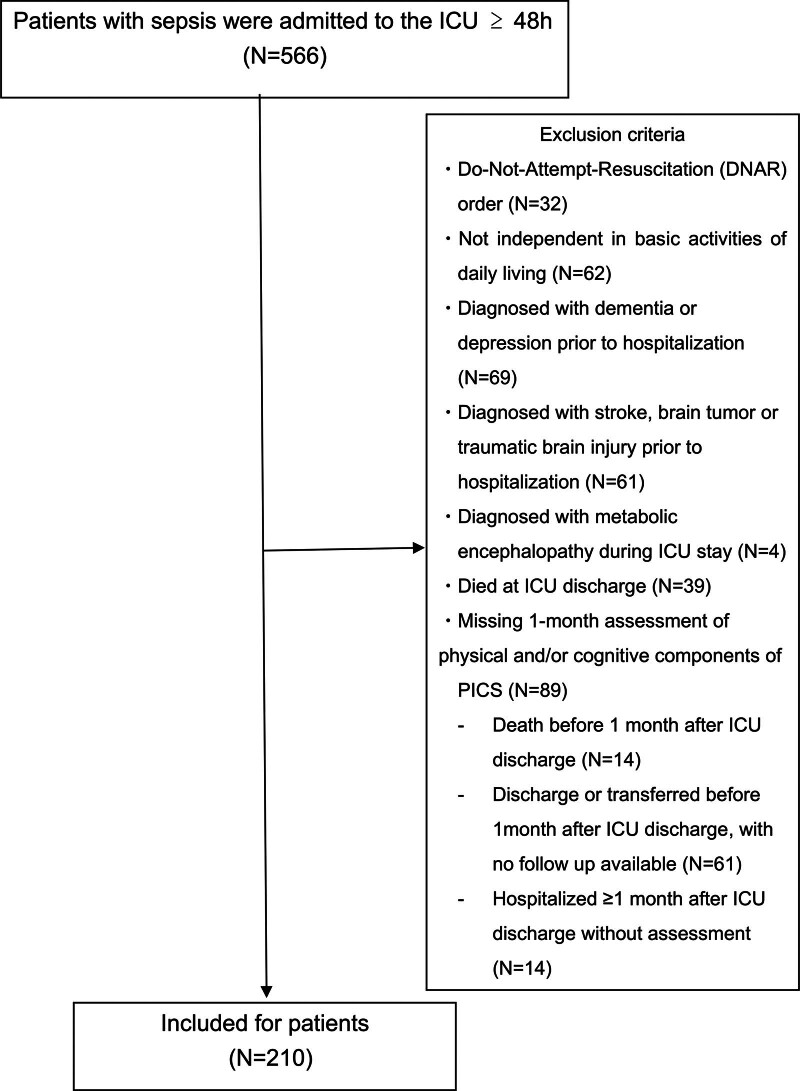
Flow chart of patient selection and exclusion process in this study.Patients without a 1-month assessment of the physical and/or cognitive components of the PICS were classified according to the reason for their missingness. ICU = intensive care unit, N = number of patients, PICS = post-intensive care syndrome.

This single-center retrospective cohort study was conducted in the ICU of Kitasato University Hospital, emergency and disaster medical center, and general ICU in Japan. Five hundred fifty-six consecutive patients aged ≥ 20 years were identified and diagnosed with sepsis based on the SEPSIS-3 criteria^[1]^ and admitted to our ICU for more than 48 hours between April 2014 and March 2021. Although “participants” is used in the section heading, the term patients are used hereafter for clarity and consistency. Patients with the following conditions were excluded from the study: Do-Not-Attempt-Resuscitation order; not independent in basic ADL or had been diagnosed with dementia, depression, stroke, brain tumor or traumatic brain injury before hospitalization; diagnosed with metabolic encephalopathy during ICU stay; died on ICU discharge; patients without a 1-month assessment of the physical and/or cognitive components of PICS, or with missing data for predefined covariates, were excluded from the analysis (Fig. 1). The study was performed in accordance with the tenets of the Declaration of Helsinki and approved by the Ethics Committee of Kitasato University Hospital (B17–086). This study included all consecutively eligible patients during the study period. The final sample size was determined on the basis of the number of patients who met the inclusion and exclusion criteria. In view of the retrospective design and the use of existing medical records, the Board waived the requirement for written informed consent. Instead, an opt-out approach was adopted; the details of this study were disclosed on the institutional website, and patients were given the opportunity to decline participation.

### 2.2. Data collection

Information on the patients’ background characteristics, including age, sex, and body mass index (BMI), clinical information, including history, acute physiology and chronic health evaluation (APACHE) II score^[18]^ at ICU admission, mean sequential organ failure assessment (SOFA) score^[19]^ during ICU stay, laboratory examination results, sedative drugs, or steroid use were collected from electronic medical records. Delirium in the ICU was evaluated using the confusion assessment method for the ICU.^[20]^ In addition, the duration of mechanical ventilation, length of ICU stay, and length of hospital stay were recorded.

### 2.3. Assessment of physical and/or cognitive PICS

Physical and cognitive impairment evaluations were performed 1 month after ICU discharge. The physical and/or cognitive components of the PICS were defined using established measures of physical and cognitive function. Physical impairment was assessed using the Barthel Index (BI),^[21,22]^ with physical impairment defined as a BI score < 85.^[21–23]^ Cognitive impairment was assessed using the Mini-Mental State Examination (MMSE), a widely used screening tool for cognitive function in hospitalized patients.^[24]^ Cognitive impairment was defined as a MMSE score < 24.^[25]^ Patients with physical and/or cognitive impairment were classified as having physical and/or cognitive components of PICS, whereas those without these impairments were classified as non-PICS. These evaluations were performed at a single time point, 1 month after ICU discharge, and were used to define the physical and/or cognitive components of PICS; no longitudinal assessments of physical or cognitive function were conducted.

### 2.4. Outcomes

We determined the survival of the patients with sepsis as the primary outcome. The secondary outcome was the identification of factors associated with the physical and/or cognitive components of the PICS. The endpoints were calculated as the number of days from ICU discharge to the date of the event occurring 1 year after ICU discharge, and all-cause death was determined by reviewing the medical records.

### 2.5. Follow-up and outcome ascertainment

Patients were followed up for up to 1 year from the date of ICU discharge. Vital status and date of death were determined by reviewing electronic medical records, referral documents, and follow-up information obtained from affiliated hospitals or primary care providers. Deaths occurring outside our hospital system were included when such information was available. Patients for whom no further information on vital status was available during the follow-up period were censored on the date of the last confirmed survival.

### 2.6. Statistical analysis

Baseline characteristics were compared using 1-way analysis of variance, Wilcoxon rank sum test, or *χ*^2^ test as appropriate. To determine the independent risk factors for the physical and/or cognitive components of PICS, multivariable analysis was performed using a binary logistic regression model to assess the occurrence of these components 1 month after ICU discharge, with clinically relevant variables. All continuous variables were analyzed as continuous measures without dichotomization for the regression analyses. Patients without a 1-month assessment of the physical and/or cognitive components of PICS, or with missing data for covariate adjustment were excluded, whereas patients lost to follow-up during the observation period were treated as right-censored in the survival analyses. The association between the physical and/or cognitive components of PICS and the primary outcome variable, all-cause mortality 1 year after ICU discharge, was examined using the Kaplan–Meier method with the log-rank test and Cox proportional hazards model. In the multivariable Cox proportional hazards models, we adjusted for preexisting risk factors including age, sex, APACHE II score, and presence of septic shock. The covariates included in the multivariable Cox proportional hazards models were selected a priori based on clinical relevance and prior literature, focusing on baseline characteristics determined before the assessment of the physical and/or cognitive components of PICS to minimize potential overadjustment. Variables related to the ICU course (including ICU length of stay, duration of mechanical ventilation, and duration of delirium) were examined in sensitivity analyses. The proportional hazards assumption was assessed for all covariates included in the Cox proportional hazards models, and no significant violations were identified. We also examined the relationships between the different components of PICS (i.e., physical and cognitive impairment) and all-cause mortality 1 year after ICU discharge using the Kaplan–Meier method with the log-rank test and multivariable Cox proportional hazard model adjusted for preexisting risk factors.

Analyses were performed using the JMP Student Edition18 (SAS Institute Inc., Cary).

## 3. Results

### 3.1. Patient characteristics

In total, 210 patients with sepsis were included in this study (Fig. 1). The characteristics of the 210 patients are summarized in Table 1. The median age of the patients was 69 years, 143 (68%) were male, and the median APACHE II score at admission was 26. To assess potential selection bias, we compared the baseline characteristics of the included and excluded patients (Table S1, Supplemental Digital Content, https://links.lww.com/MD/R722). The groups were broadly comparable in age, BMI, and severity indicators (septic shock, APACHE II, and mean daily SOFA scores), whereas the proportion of males differed between the groups. Missing 1-month assessments were mainly attributable to early discharge/transfer, death within 1 month after ICU discharge, or incomplete assessments despite prolonged hospitalization (Fig. 1). Patients were classified into 2 groups according to the presence of physical and/or cognitive components of PICS at 1 month after ICU discharge: a component-positive group (N = 99, 47%) and a component-negative group (N = 111, 53%). Univariate analyses were performed to identify risk factors associated with the occurrence of these components between the 2 groups. In the univariate survival analysis, 12 variables, including age (*P* < .001), APACHE II score at admission (*P* < .001), mean daily SOFA score (*P* = .011), cause of sepsis (others) (*P* = .002), ICU length of stay (*P* < .001), length of hospital stay (*P* < .001), duration of mechanical ventilation (*P* < .001), presence of delirium (*P* < .001), duration of delirium (*P* < .001), procalcitonin at admission (*P* < .046), prealbumin (*P* < .029), and 1-year mortality after ICU discharge (*P* < .001) were statistically different between the 2 groups (Table 1).

**Table 1:** Characteristics, treatment, outcomes, assessments of physical and/or cognitive components PICS among the study patients

	All patients	Physical and/or cognitive PICS at 1 month	No physical or cognitive PICS at 1 month	*P* value
	(N = 210)	(N = 99)	(N = 111)	
Age, years Males sex, n (%) BMI (kg/m^2^)	69 (56–76) 143 (68) 23.1 (19.9–25.8)	74 (64–79) 65 (65.7) 22.8 (19.1–26.1)	65 (52–73) 78 (70.3) 23.2 (20.5–25.6)	< .001* .47 .24
Severity, n (%) Septic shock at admission APACHE II score at admission Mean daily SOFA score	172 (81.9) 26 (20.5–32) 8 (5–10)	85 (85.9) 28 (23–34.5) 9 (6–11)	87 (78.4) 24 (18–30) 7 (4.4–10)	.16 < .001* .011*
Comorbidities, n (%) Hypertension Diabetes Cancer	73 (34.5) 61 (29) 72 (34.3)	41 (41.4) 26 (26.3) 37 (37.4)	32 (28.8) 35 (31.5) 35 (31.5)	.06 .41 .37
Causes of sepsis, n (%) Pneumonia Intra-abdominal infection Urinary Tract Infection Soft tissue infection Others	41 (19.5) 119 (56.7) 24 (11.4) 14 (6.7) 12 (4.7)	23 (23.2) 54 (54.5) 13 (13.1) 8 (8.1) 1 (1.1)	18 (16.2) 65 (58.6) 11 (9.9) 6 (5.4) 11 (9.9)	.21 .56 .46 .44 .002*
Present of operation, n (%)	111 (53.1)	48 (48.9)	63 (56.8)	.26
ICU length of stay (d) Hospital length of stay (d)	8 (6–14) 33 (20–60.3)	11 (7–18) 45 (21–78)	7 (5–10) 28 (19–49)	< .001* < .001*
Mechanical ventilation Patients, n (%) Duration of ventilation, days	131 (62.4) 2 (0–7)	65 (65.7) 4.5 (0–12)	66 (59.5) 2 (0–4)	.35 < .001*
CVVHDF, n (%)	94 (44.8)	46 (46.5)	48 (43.2)	.64
Delirium Patients, n (%) Duration of delirium, days	137 (65.2) 2 (0–4.3)	84 (84.9) 4 (2–6)	53 (47.8) 0 (0–2)	< .001* < .001*
Sedative Midazolam use Treated, n (%) Dose, mg/day Propofol use Treated, n (%) Dose, µg/kg/min Dexmedetomidine use Treated, n (%) Dose, µg/kg/h	80 (38) 42.9 (19.2–76) 99 (47.1) 8.1 (4.4–12.5) 104 (49.5) 0.2 (0.1–0.3)	44 (44.4) 40.8 (17.7–66.6) 49 (49.9) 9.2 (3.9–13.8) 55 (55.6) 0.2 (0.1–0.32)	36 (32.4) 47.3 (21–101) 50 (45.1) 7.6 (4.6–11.7) 49 (44.1) 0.2 (0.1–0.29)	.07 .42 .52 .87 .098 .85
Steroid use, n (%)	64 (30.5)	32 (32.3)	32 (28.8)	.58
Laboratory examination CRP at admission, mg/l PCT at admission, ng/ml PA at admission, mg/l	12.1 (3.4–26.2) 8.8 (1.1–47.2) 9 (6–12)	13.1 (4.4–27.9) 10.9 (1.7–60.8) 8 (5–11)	11.6 (2.3–21.8) 3.9 (0.6–38.1) 10 (6–14)	.15 .046* .029*
Physical and/or cognitive PICS assessments at 1 month Physical impairment, n (%) BI score Cognitive impairment, n (%) MMSE score	96 (45.7) 85 (50–90) 59 (28.1) 28 (25–30)	96 (96.9) 50 (15–65) 59 (59.6) 25 (20–27)	0 (0) 90 (85–95) 0 (0) 29 (28–30)	< .001* < .001* < .001* < .001*
1-year mortality after ICU discharge, n (%)	43 (20.5)	41 (41.4)	2 (1.8)	< .001*

Values are Median (Interquartile range) or % of total.

APACHE II = Acute Physiology and Chronic Health Evaluation II, BMI = body mass index, CRP = C-reactive protein, CVVHDF = Continuous veno-venous hemodiafiltration, ICU = intensive care unit, MMSE = Mini-mental state examination, N = number of patients, PA = Prealbumin, PCT = Procalcitonin, PICS = post-intensive care syndrome, SOFA = Sequential Organ Failure Assessment.

*: *P* < .05.

### 3.2. Identification of risk factors related to physical and/or cognitive PICS

**Figure 2.**
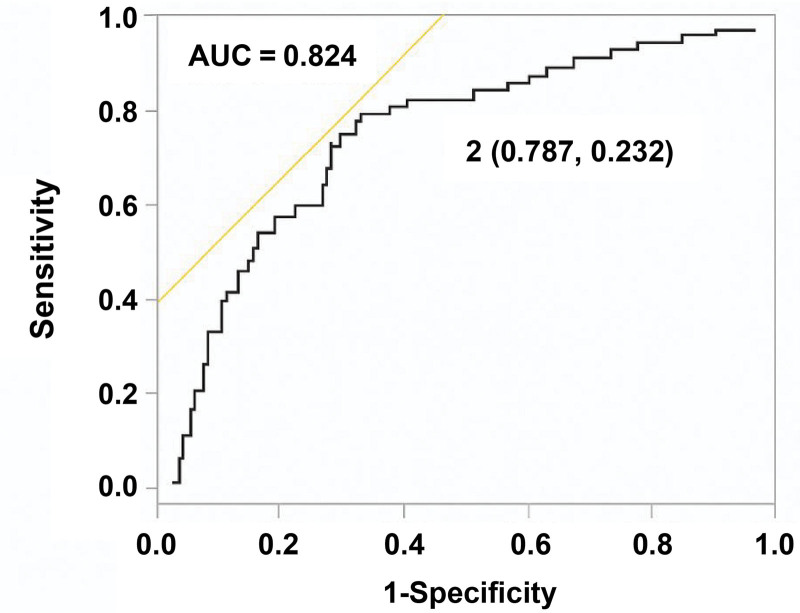
Receiver Operating Characteristic Curve of delirium duration for Predicting the physical and/or cognitive components of PICS at 1 month after ICU discharge. ROC curves showing the AUC and cutoff points for the duration of delirium associated with the physical and/or cognitive components of PICS at 1 month after ICU discharge. AUC = area under the curve, ICU = intensive care unit, PICS = post-intensive care syndrome, ROC = receiver operating characteristic curve.

To explore the risk factors for the physical and/or cognitive components of the PICS, we conducted a logistic regression analysis with clinically valid variables. After adjusting for age, sex, BMI, APACHE II score, and the presence of septic shock, increasing age (odds ratio [OR] 1.05, 95% confidence interval [CI] 1.02–1.09; *P* < .001), longer ICU stay (OR 1.09, 95% CI 1.01–1.17; *P* = .031) and prolonged delirium (OR 1.41, 95% CI 1.19–1.67; *P* < .001) were independent predictors of the occurrence of physical and/or cognitive PICS. For physical impairment, increasing age (OR 1.05, 95% CI 1.02–1.08; *P* < .001), longer ICU stay (OR 1.09, 95% CI 1.01–1.18; *P* = .021) and prolonged delirium (OR 1.33, 95% CI 1.14–1.57; *P* < .001) were independent predictors. For cognitive impairment, increasing age (OR 1.05, 95% CI 1.01–1.08; *P* = .008), male sex (OR 1.35, 95% CI 1.13–1.54; *P* = .038), longer ICU stay (OR 1.10, 95% CI 1.02–1.19; *P* = .016), and prolonged delirium (OR 1.23, 95% CI 1.05–1.44; *P* = .008) were independent predictors (Table 2). Receiver operating characteristic curve analysis demonstrated that the duration of delirium was a significant predictor of the physical and/or cognitive components of PICS. In this exploratory analysis, a delirium duration of 2 days was identified as the optimal cutoff value, with a sensitivity of 78.7% and a specificity of 76.8% (Fig. 2).

**Table 2:** Logistic regression analysis for predicting physical and/or cognitive components of PICS at 1 month after ICU discharge.

	Physical and or cognitive components of PICS			Physical impairment			Cognitive impairment		
	BI < 85 or MMSE < 24			BI score < 85			MMSE score < 24		
Variables	OR	95%CI	*P* value	OR	95%CI	*P* value	OR	95%CI	*P* value
Age Male BMI (per 1.0 kg/m^2^) APACHE Ⅱ score Septic shock at admission PCT at admission Duration of delirium (per 1d) Duration of ventilation (per 1 d) ICU length of stay (per 1 d)	1.05 1.23 0.99 0.98 1.02 1.00 1.41 1.01 1.09	1.02–1.09 0.99–1.41 0.92–1.06 0.93–1.03 0.81–1.23 0.99–1.01 1.19–1.67 0.93–1.12 1.01–1.17	< .001* .38 .72 .42 .96 .65 < .001* .83 .03*	1.05 1.21 0.99 0.98 1.03 1.00 1.33 1.01 1.09	1.02–1.08 0.99–1.39 0.92–1.06 0.93–1.03 0.81–1.21 0.99–1.01 1.14–1.57 0.93–1.11 1.01–1.18	< .001* .43 .78 .41 .86 .54 < .001* .81 .02*	1.05 1.35 0.96 0.97 1.02 1.00 1.23 1.05 1.10	1.01–1.08 1.13–1.54 0.88–1.04 0.91–1.02 0.81–1.23 0.99–1.01 1.05–1.44 0.97–1.17 1.02–1.19	.008* .038* .28 .22 .96 .68 .008* .24 .016*

Continuous variables were analyzed as continuous covariates in the logistic regression model.

APACHE II = Acute Physiology and Chronic Health Evaluation II, BI = Barthel Index, BMI = body mass index, CI = confidential interval, ICU = intensive care unit, MMSE = Mini-Mental State Examination, OR = Odds Ratio, PCT = Procalcitonin.

*: *P* < .05.

### 3.3. Association of physical and/or cognitive PICS after sepsis with all-cause 1-year mortality

**Figure 3.**
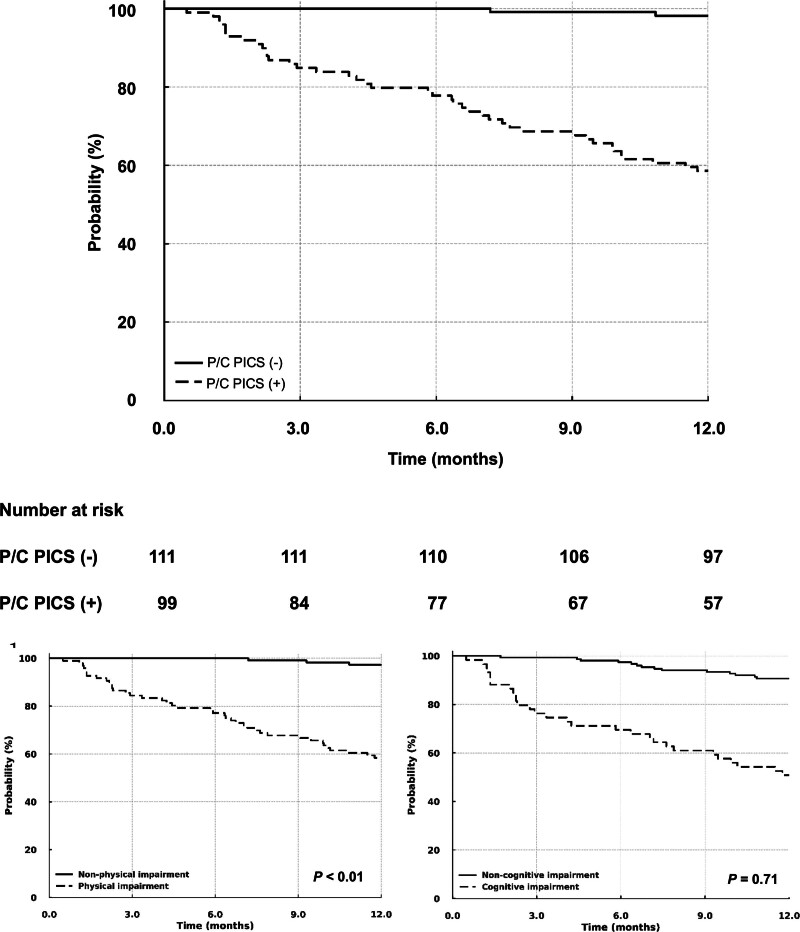
Kaplan–Meier analyses for 1-year outcomes in patients with sepsis according to the presence of the physical and/or cognitive components of PICS, physical impairment, and cognitive impairment at 1 month after ICU discharge. P/C PICS indicates the presence of physical and/or cognitive components of PICS at 1 month after ICU discharge. Physical impairment was defined as a BI score of < 85, and cognitive impairment was defined as an MMSE score of < 24. BI = Barthel Index, ICU = intensive care unit, P/C = physical and/or cognitive components of PICS, PICS = post-intensive care syndrome.

To address the impact of physical and/or cognitive PICS 1 month after ICU discharge on long-term outcomes, we compared 1-year survival after sepsis between patients with and without physical and/or cognitive PICS. The 1-year mortality rate after ICU discharge among the study patients was 20% (Table 1). During the 1-year follow-up period, 13 patients were lost to follow-up and were censored at their last confirmed date of survival. Kaplan–Meier analysis indicated that patients with physical and/or cognitive PICS had a significantly higher mortality rate than those without physical and/or cognitive PICS (log-rank test: *P* < .01). In the multivariable Cox regression analyses, physical and/or cognitive PICS were also significantly associated with all-cause 1-year mortality after adjusting for covariates (adjusted hazard ratio [HR] 1.51, 95% CI 1.06–2.15; *P* = .021). Regarding the associations between physical or cognitive impairment and mortality, physical impairment 1 month after ICU discharge was significantly associated with a higher rate of all-cause 1-year mortality in the Kaplan–Meier analysis (log-rank test: *P* < .01). However, cognitive impairment 1 month after ICU discharge was not significantly associated with mortality (log-rank test: *P* = .71) (Fig. 3). In the multivariable Cox proportional hazards model, physical impairment was significantly associated with all-cause 1-year mortality after adjusting for covariates (HR 1.52, 95% CI 1.07–2.17; *P* = .019), but cognitive impairment was not significantly associated with all-cause 1-year mortality (HR 1.48, 95% CI.96–2.29; *P* = .073). In sensitivity analyses additionally adjusting for ICU course–related variables (ICU length of stay, duration of mechanical ventilation, and duration of delirium; Model 2) and recorded comorbidities (hypertension, diabetes, and cancer; Model 3), the HRs for the physical and/or cognitive components of PICS remained directionally consistent with those observed in the primary model, although the associations were attenuated and no longer statistically significant. The results of these analyses are presented in Table S2, Supplemental Digital Content, https://links.lww.com/MD/R722.

## 4. Discussion

This study demonstrated that, among 210 patients with sepsis, the 1-year survival rate was 79.5, and 47% of the patients exhibited physical and/or cognitive impairment 1 month after ICU discharge. Increasing age, prolonged delirium during ICU stay, and longer ICU stay were independently associated with the development of physical and/or cognitive components of PICS. In addition, sepsis survivors with physical and/or cognitive impairment 1 month after ICU discharge had a lower 1-year survival rate than those without these components. Among these components, physical impairment was associated with 1-year mortality, while cognitive impairment was not.

Patients with physical and/or cognitive PICS had a longer duration of delirium than those without. Furthermore, prolonged delirium was an independent prognostic factor for the physical and/or cognitive components of PICS at 1 month after ICU discharge. Patients who had delirium in the ICU more often reported problems in ADL and worse sensorimotor function test scores at long-term follow-up.^[26,27]^ Delirium during sepsis is considered a diffuse brain dysfunction resulting from a systemic inflammatory response to infection^[28]^ and is associated with long-term cognitive impairment, mainly affecting memory, attention, and verbal fluency.^[9,29]^ Attention impairment is correlated with motor function disorders and falls.^[30]^ Although early mobilization in the ICU environment has the potential to prevent or minimize dysfunction in critically ill patients,^[31,32]^ Bennion et al reviewed the presence of delirium was the most commonly reported barrier to early mobilization.^[33]^ Delayed initiation of early mobilization leads to prolonged bed rest and immobilization, which may result in complications such as muscular wasting, and ICU-acquired weakness,^[34–36]^ ultimately leading to impaired mobility and performance of ADL and an overall functional decline.^[37]^ Taken together, these previous reports and the findings of the present study suggest that among patients with sepsis, prolonged delirium has been suggested to predict not only cognitive impairment but also subsequent physical impairment after ICU discharge, potentially compromising functional independence in ADL.

In the present study, an receiver operating characteristic-derived cutoff value of 2 days for the duration of delirium showed relatively high sensitivity (78.7%) and specificity (76.8%) for predicting the physical and/or cognitive components of PICS. However, this cutoff value was derived from an exploratory, single-center analysis and should be interpreted as hypothesis-generating rather than as a definitive clinical threshold. External validation in independent cohorts is required before this value can be applied to clinical practice. Nonetheless, our findings suggest that prolonged delirium during ICU stay may serve as an important marker of vulnerability to subsequent PICS, highlighting the importance of careful post-ICU follow-up.

In this study, physical impairment, defined as a decline in ADL independence, was independently associated with an increased risk of 1-year mortality. The development of physical dysfunction after sepsis is thought to involve multifactorial pathophysiology, including systemic inflammatory responses, immune dysregulation, microcirculatory impairment, mitochondrial dysfunction, and neuromuscular junction abnormalities.^[38–40]^ In addition, several risk factors, including age, comorbidities, illness severity, organ failure, exposure to medications negatively affecting myofibers and neurons, and immobility and other intensive care-related factors, have been identified as major contributors to physical functional decline.^[41]^ The complex interplay among these factors may exacerbate muscle wasting, leading to intensive ICU-acquired weakness or severe sarcopenia-like condition. Moreover, low muscle mass after sepsis has been independently associated with increased 1-year mortality.^[42]^ These findings suggest that physical impairment after ICU discharge in sepsis survivors is not merely a functional limitation but may significantly affect long-term outcomes, including mortality risk. However, given the observational nature of this study, residual confounding is likely, and the observed association should not be interpreted as causal or reflecting a unidirectional pathway from functional decline to death. Unmeasured factors such as baseline frailty, pre-ICU functional reserve, and comorbidities may have influenced both post-ICU physical impairment and subsequent mortality risk, and the causal mechanisms underlying this relationship remain unclear. The generalizability of these findings should be interpreted with caution. The analytic cohort comprised sepsis survivors without preexisting disability, dementia, or diagnosed depression, representing a relatively preserved population prior to ICU admission. Thus, the observed association may not be directly applicable to older or frail patients, in whom baseline vulnerability may substantially modify recovery trajectories and long-term outcomes. Moreover, as this was a single-center study conducted in a Japanese university hospital ICU, differences in case-mix, sedation and delirium management, early rehabilitation practices, and post-discharge care systems may further limit the applicability to Western ICU populations or other healthcare settings. Accordingly, future studies should longitudinally assess changes in muscle mass, muscular strength, and neuromuscular function, and investigate their associations with mortality and quality of life using multivariable analytical approaches in more heterogeneous and multicenter cohorts to improve causal inference and external validity.

This study has several limitations. First, although we performed sensitivity analyses with additional adjustment for ICU course variables and recorded comorbidities, residual confounding is likely due to the observational design, and the observed associations should not be interpreted as causal. In particular, unmeasured factors such as frailty, pre-ICU functional status, and other comorbid conditions not captured in our dataset may have influenced both the development of the physical and/or cognitive components of PICS and long-term mortality. Second, this was a single-center retrospective study conducted in a Japanese university hospital ICU, which may limit the generalizability of our findings to other ICU settings, healthcare systems, or patient populations. Third, the analytic cohort differed from the excluded patients in terms of sex distribution, and patients who died before ICU discharge or before the 1-month assessment were excluded. Consequently, the generalizability of our findings may be limited, and the 1-year mortality estimates may have been underestimated owing to survivor bias. Fourth, we evaluated PICS using a limited number of questionnaires, including the BI and MMSE. Fifth, delirium was assessed only during ICU stay; therefore, the potential impact of persistent or recurrent delirium after ICU discharge on subsequent physical or cognitive impairment could not be evaluated. Finally, we did not collect data on mental health measures, such as depression, anxiety and post-traumatic stress disorder, which may also influence long-term functional outcomes and mortality in sepsis survivors.

## 5. Conclusions

Increasing age, prolonged delirium, and longer ICU stay were independently associated with the development of the physical and/or cognitive components of PICS 1 month after ICU discharge. Among the sepsis survivors evaluated at this time point, the presence of these components was independently associated with higher 1-year mortality in this cohort, with physical impairment showing a significant association with increased 1-year mortality.

## Acknowledgments

We would like to thank the patients and the research team for their participation in this study.

The statistical analyses were conducted under the supervision of Dr Hirokuni Tagaya

## Author contributions

**Conceptualization:** Michiteru Miyazaki.

**Data curation:** Michiteru Miyazaki, Hirokuni Tagaya.

**Formal analysis:** Michiteru Miyazaki, Hirokuni Tagaya.

**Investigation:** Michiteru Miyazaki.

**Methodology:** Michiteru Miyazaki, Hirokuni Tagaya.

**Project administration:** Michiteru Miyazaki.

**Resources:** Yuichi Kataoka, Masayasu Arai.

**Software:** Michiteru Miyazaki.

**Supervision:** Hirokuni Tagaya.

**Validation:** Michiteru Miyazaki.

**Visualization:** Michiteru Miyazaki.

**Writing – original draft:** Michiteru Miyazaki.

**Writing – review & editing:** Hirokuni Tagaya, Kazumasa Miida, Hidenori Kariya, Yuichi Kataoka, Masayasu Arai.

## Supplementary Material


