# Supplementary material for: Association between risk factors and 1-year mortality of physical and/or cognitive components of post-intensive care syndrome in patients with sepsis
Source: Medicine (Baltimore). 2026 Apr 17;105(16):e48425. doi: 10.1097/MD.0000000000048425 (PMC13095336; doi:10.1097/MD.0000000000048425)
Supplement: Supplementary file 1 [file medi-105-e48425-s001.pdf]

Supplementary Table S1. Comparison of baseline characteristics between included and excluded patients.

	All eligible patients	Included	Excluded	P value
	(N=566)	(N=210)	(N=356)	
Age, years	70 (60.3–77)	69 (56–76)	71 (61.7–77)	.07
Males sex, n (%)	354 (62.5)	143 (68)	211 (59.3)	.04*
Body mass index (kg/m^2)	22.6 (19.7–25.8)	23.1 (19.9–25.8)	22.3 (19.6–25.8)	.35
Severity				
Septic shock at admission, n (%)	478 (84.5)	172 (81.9)	306 (85.9)	.22
APACHE II score at admission	26 (20–31.7)	26 (20.5–32)	25 (20–31)	.85
Mean daily SOFA score	8 (5–11)	8 (5–10)	8 (5–11)	.63

Values are Median (Interquartile range) or % of total. *: $p < 0.05$

APACHE II = Acute Physiology and Chronic Health Evaluation II, SOFA = Sequential Organ Failure Assessment.

Supplementary Table S2. Sensitivity analyses of the association between the physical and/or cognitive components of PICS and 1-year all-cause mortality using multivariable Cox proportional hazards models

Physical and/or cognitive components of PICS (any component)			
Model	Hazard ratio (HR)	95% Confidence interval	P value
Model 1	1.51	1.06–2.15	.021
Model 2	1.27	0.84–1.91	.25
Model 3	1.29	0.85–1.96	.22
Physical impairment			
Model	Hazard ratio (HR)	95% Confidence interval	P value
Model 1	1.52	1.07–2.17	.019
Model 2	1.3	0.87–1.94	.21
Model 3	1.31	0.87–1.97	.19
Cognitive impairment			
Model	Hazard ratio (HR)	95% Confidence interval	P value
Model 1	1.48	0.96–2.29	.073
Model 2	1.12	0.64–1.95	.69

Model 3	1.27	0.72–2.25	.39

Model 1 was adjusted for age, sex, APACHE II score, and presence of septic shock.

Model 2 was additionally adjusted for ICU length of stay, duration of mechanical ventilation, and duration of delirium.

Model 3 was further adjusted for recorded comorbidities (hypertension, diabetes, and cancer).

These models were conducted as sensitivity analyses to assess the robustness and directionality of the associations rather than statistical significance.

APACHE II = Acute Physiology and Chronic Health Evaluation II, PICS = post-intensive care syndrome.
